# Partial vertebrectomy with spine shortening for old spondyloptosis at the thoracolumbar spine: a case series study and literature review

**DOI:** 10.3389/fsurg.2023.1206395

**Published:** 2023-07-21

**Authors:** Nuo Xu, Ping Liu, Yijun Kang, Fei Chen

**Affiliations:** Department of Spine Surgery, The Second Xiangya Hospital, Central South University, Changsha, China

**Keywords:** complete fracture-dislocation, spondyloptosis, partial vertebrectomy, spinal shortening, thoracolumbar spine

## Abstract

**Objective:**

We aimed to report the surgical outcomes of serial cases and retrospectively analyze the value of partial vertebrectomy and spinal shortening in the reduction of old spondyloptosis at the thoracolumbar spine.

**Methods:**

From 2015 to 2021, eight cases of patients who received a spinal intervention of partial vertebrectomy and spinal shortening for thoracolumbar spondyloptosis over 3 weeks post-trauma were retrospectively summarized. Medical records and surgical outcomes were extracted for clinical safety and efficacy evaluation.

**Results:**

Acceptable reduction and immediate stabilization were achieved for all eight cases without causing iatrogenic damage to the viscera. The mean operation time was 3.7 h (range, 3.2–4.2 h) with a mean blood loss average of 1,081 ml (range, 900–1,300 ml). Postoperative stay in the spine department was an average of 11.4 days (range, 8–17 days), followed by an early rehab program. The mean visual analog scale (VAS) for low back pain decreased from 8.0 preoperatively to 1.4 at the last follow-up. The average follow-up period was 19.9 months. As for neurological function recovery, six patients with preoperative ASIA-A status remained unchanged throughout the follow-up period and improvement of one ASIA grade was noted in two patients. At the latest follow-up, sound interbody fusion as well as good alignment of the spinal column were confirmed radiologically in seven patients, while one patient encountered slight re-dislocation 3 months after surgery, but eventually achieved spinal fusion.

**Conclusion:**

Partial vertebrectomy and spine shortening via a posterior approach showed good efficacy and safety in the management of old spondyloptosis of the thoracolumbar spine, allowing for a one-step good reduction and spinal fusion for early rehabilitation.

## Introduction

Complete fracture-dislocation, termed spondyloptosis, at the thoracolumbar segments is a rare but severe traumatic event with a high likelihood of neurologic deficit (1). Timely surgery within days for the decompression of neural elements and the restoration of spine alignment is critical for a potential chance of neural recovery and personal mobilization (2). Unfortunately, complete fracture-dislocation happens concomitantly with multisystem injuries due to high-energy trauma, which often medically precludes patients from immediate spinal intervention. Subsequently, the lesions around fracture sites developed old and became even more complex over time. Whenever possible, a delayed intervention for the correction of this post-traumatic deformity is necessary, regardless of neurological deficit, to pursue early rehabilitation and better quality of life (3). However, effectively and safely reducing old spondyloptosis, especially when it has developed over 3 weeks, poses a challenging scenario for the spinal surgeon.

There are sporadic reports available in English that have described surgical strategies in the management of thoracolumbar spondyloptosis (4). However, for old fracture-dislocation with lesion scarring and abnormal bone healing around the fracture site, a direct reduction with intraoperative distraction becomes difficult and impossible, thus necessitating a spinal shortening procedure. In recent years, total vertebrectomy-spine shortening, deemed as the last surgical technique reserved for persistent spinal deformities, was introduced to facilitate the reduction of complete fracture-dislocation and show its feasibility (5–7). Nonetheless, total vertebrectomy appeared overaggressive when factoring in the patient's surgical tolerance in the debilitating state after a deadly trauma (8). In such a context, the technique of vertebrectomy has to be modified as less invasive while pursuing spine shortening.

In 2002, Reyes-Sanchez et al. (9) described a technique of partial vertebrectomy in the treatment of burst fractures, resulting in a high spinal fusion rate. This technique basically consisted of resection of the proximal proportion of the fractured vertebra as well as the posterior arch and upper disc. By doing so, the spinal shortening was completed and the intraoperative distraction and reduction could be manipulated. To our knowledge, the technique of modified partial vertebrectomy in the chronic phase of thoracolumbar spondyloptosis has never been reported. Hence, we reported the surgical outcomes of a series of cases and retrospectively analyzed the value of partial vertebrectomy and spine shortening in the reduction of old spondyloptosis.

## Materials and methods

From 2015 to 2021, eight patients underwent spinal surgery for complete thoracolumbar fracture-dislocation at our department, which was delayed for over 3 weeks since the trauma. Among them, five first arrived at the emergency department of our hospital and immediately received multidisciplinary care for concomitant life-threatening visceral injuries. When their vital signs stabilized, they were transferred to our department for further spinal intervention. The remaining three cases were referred to our department from the local hospitals. A preoperative CT reconstruction and MRI scans were performed for all patients to help with decision-making. X-rays (anteroposterior and lateral views) were routinely taken to observe spinal alignment. Medical records and surgical outcomes were retrospectively analyzed and reviewed. This prospective study was approved by the research and ethics committee of our hospital and written informed consent was obtained from each of the participants.

The delayed spinal intervention for all patients was performed by the same medical team led by the corresponding author, a senior spine surgeon. The surgical procedures generally consisted of long-segment instrumentation (at least two levels below and two above the fracture site), partial vertebrectomy-spine shortening, reduction, fixation as well as interbody fusion, via a posterior one-stage approach. A partial vertebrectomy of the fractured vertebra was carried out in reference to the description by Reyes-Sanchez (9). Physical examinations and radiographies were routinely performed at 3, 6, 12, and 24 months after surgery. The mean follow-up period was 19.9 months (range, 12–24 months).

## Results

The baseline characteristics of the eight patients (six men, two women; mean age, 28.3 years; age range, 19–42 years) are presented in Table 1. The leading cause of trauma was traffic accident (four cases), followed by fall from height (three cases), and mine collapse (one case). Concomitant life-threatening visceral injuries included hemopneumothorax with a severe chest injury, coma with a head injury, splenic lacerations, kidney rupture, sepsis, and peritonitis with a pancreas injury or hepatic rupture, which precluded patients from timely spinal intervention. The mean interval between trauma and surgery was 33.5 days (range, 25–45 days). In the sagittal plane, six patients presented dislocation of the upper vertebral body anterior to the caudal vertebra, whereas two presented posterior spondyloptosis. The dislocation levels among the patients were distributed as follows: T11–12 in three patients; T12–L1 in four patients; and L1–2 in one patient. The severity of back pain was evaluated using a visual analog scale (VAS) in the range of 0–10. Neurological function status was assessed and graded using the American Spinal Injury Association (ASIA) impairment scale. The grade of neurological status on admission was distributed as follows: ASIA-A in seven patients; and ASIA-B in one patient.

**Table 1:** Baseline characteristics of 8 patients with old thoracolumbar spondyloptosis.

Patient	Age/sex	Trauma	Dislocation level	Interval^a^ (days)	Reason for delay of operation	Preoperative VAS of low back pain	Preoperative ASIA grade	Follow-up (months)
1	32/F	Fall from height	T12–L1	31	Sepsis with hemopneumothorax	9	A	15
2	42/M	Traffic accident	T11–12	25	Coma with a head injury	6	A	24
3	26/M	Fall from height	T11–12	40	Sepsis with hemopneumothorax and splenic lacerations	8	A	24
4	19/M	Traffic accident	T12–L1	38	Sepsis after peritonitis with hepatic rupture	9	A	12
5	29/F	Traffic accident	T12–L1	26	Peritonitis with pancreas injury	9	A	18
6	25/M	Traffic accident	T12–L1	45	Hemopneumothorax with a chest injury	7	A	24
7	33/M	Mine collapse	T11–12	26	Hemopneumothorax and kidney rupture	9	B	24
8	20/M	Fall from height	L1–2	37	Peritonitis with hepatic rupture	7	A	18

VAS, Visual Analog Scale; ASIA, American Spinal Injury Association.

^a^ Time duration between surgery and trauma.

The surgical outcomes are summed up in Table 2. Acceptable sagittal and coronal reduction and immediate stabilization were achieved for all eight cases. The mean operation time was 3.7 hours (range, 3.2–4.2 h) with a mean blood loss of 1,081 ml (range, 900–1,300 ml). Notably, no iatrogenic damage to the adjacent viscera occurred. Postoperatively, all patients experienced a smooth recovery, except patient 4 who experienced a surgical site infection that was suppressed by enhanced antibiotics. The mean postoperative stay at our department was 11.4 days (range, 8–17 days), followed by an early rehab program for all cases. The VAS for low back pain decreased from the preoperative mean score of 8.0 to 1.4 at the last follow-up. As for neurologic function recovery, six patients with preoperative ASIA-A status remained unchanged throughout the follow-up period while an improvement of one ASIA grade was noted in two patients. Patient 2 regained partial bladder-bowel function from preoperative paraplegia and patient 7 obtained deep sensation and approximate two-level muscle strength from their preoperative ASIA-B status. At the latest follow-up, sound interbody fusion as well as good alignment of the spinal column were confirmed radiologically in seven patients, while one patient (patient 7) encountered a slight re-dislocation resulting from a screw loosening at 3 months after surgery, but eventually achieved spinal fusion.

**Table 2:** Surgical outcomes of 8 patients undergoing partial vertebrectomy with spinal shortening.

Patient	Operation time (hours)	Blood loss (ml)	Iatrogenic visceral injury	Postoperative stay days at the spine department (days)	VAS of low back pain		ASIA grade		Realignment^a^	Solid spinal fusion
					Preoperative	Postoperative^a^	Preoperative	Postoperative^a^		
1	3.5	1,200	No	11	9	2	A	A	Sustained	Yes
2	4.1	900	No	14	6	1	A	B	Sustained	Yes
3	3.2	950	No	9	8	1	A	A	Sustained	Yes
4	3.6	1,100	No	17	9	1	A	A	Sustained	Yes
5	4.2	1,300	No	12	9	2	A	A	Sustained	Yes
6	3.6	1,250	No	8	7	0	A	A	Sustained	Yes
7	3.8	1,050	No	9	9	3	B	C	Slight re-dislocation	Yes
8	3.5	900	No	11	7	1	A	A	Sustained	Yes

VAS, Visual Analog Scale; ASIA, American Spinal Injury Association.

^a^ Data of the last follow-up.

## Illustrative case

**Figure 1:**
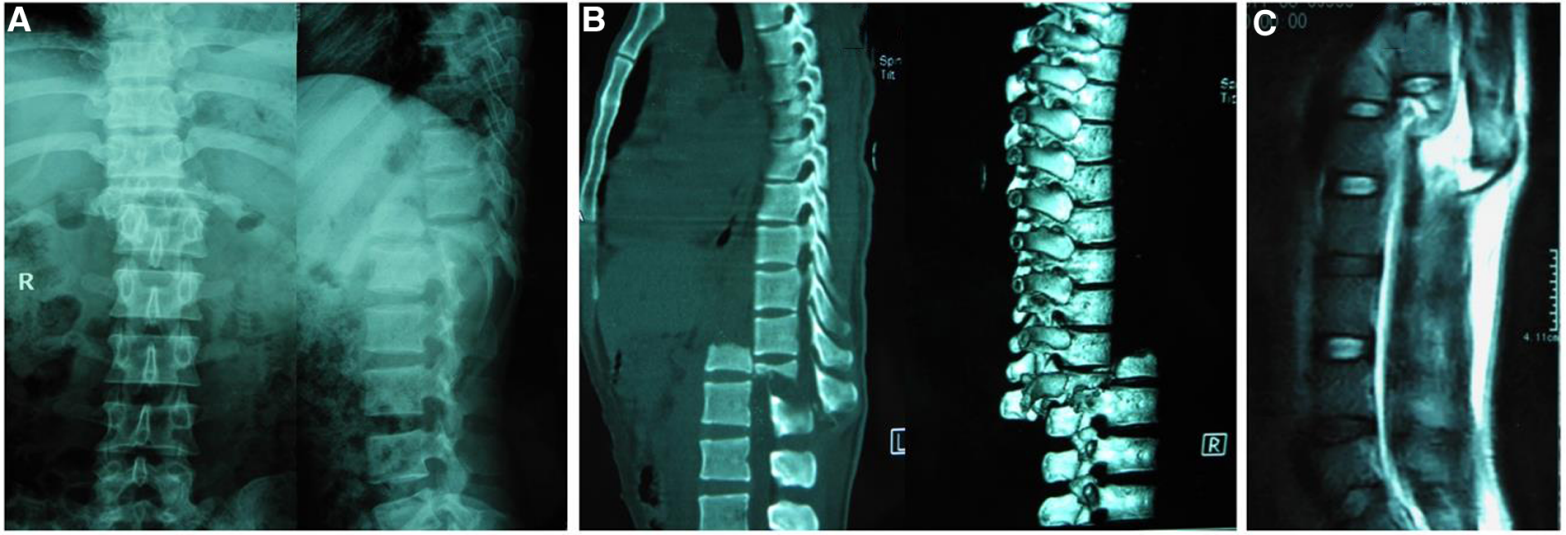
Preoperative x-ray (**A**) and reconstruction 3D CT (**B**) depicted complete posterior spondylopotosis of the upper 1/3 T12 overriding to the lower part of T12. Sagittal T2-weighted MRI (**C**) depicted a completely distorted thecal sac and spinal cord.

A 26-year-old man fell from a five-storey building, immediately experiencing severe thoracic pain and a loss of leg function. An emergency radiological examination revealed an overriding of upper T12 fragments posterior to the lower part of T12 with apparent spinal shortening and complete disruption of the thecal sac as evidenced on X-ray (Figure 1A), CT scanning and reconstruction (Figure 1B), and MRI imaging (Figure 1C). Unfortunately, the patient also developed pneumonia and sepsis after his multisystem injuries, which prevented him from undergoing spinal intervention. The patient was shifted to the spinal surgery department 35 days post-trauma until his vital signs stabilized. A preoperative specialized physical examination presented with a palpable step at the thoracolumbar junction as well as flaccid paraplegia with a sensory level of T12, a complete neurological deficit of lower extremities, and bladder-and-bowel dysfunction (ASIA Grade A). The VAS score for low back pain was eight. At this point, open reduction was still deemed necessary and tolerable for the patient. A preoperative discussion suggested that a partial vertebrectomy and spinal shortening with instrumentation to reduce the dislocation via a posterior approach was the most appropriate strategy according to the patient's comprehensive condition.

A total of 40 days after the trauma, surgical treatment for this old spondyloptosis was performed. Explicitly, via a standard posterior midline approach, a subperiosteal dissection from T10–L2 was carried out laterally to expose the tips of the transverse processes with caution at the fracture-dislocation. Meticulous lysis of the adhesions and debridement of the mobile bone fragments at the old lesion were carried out to give a good surgical field. After a total laminectomy across T11–T12, exquisite lysis of scarring adhesions along the spinal canal was performed to visualize the underlying thecal sac, which was remarkably distorted across the fracture-dislocation and lacerated by bone fragments. Furthermore, lateral decompression allowed the identification of T11 and T12 nerve roots, finding T11 nerve roots intact while T12 roots were truncated. After sparing the destroyed neural tissue thoroughly, the subsequent distraction became safe with clear visualization. At this point, the goal was the restoration of the anatomical alignment of the spine. Under fluoroscopic guidance, eight pedicle screws with long tails were inserted into T10–L2. A temporary rod was inserted transversely between the pedicle screws at the levels above and below the dislocation. The screw caps were tightened provisionally and then used as grips to manipulate the two ends for reduction. Despite applying forceful distraction, the movability of the dislocation space was minimal, and the direct reduction was impossible to complete since chronic scarring and adhesions got in the way of the distraction.

**Figure 2:**
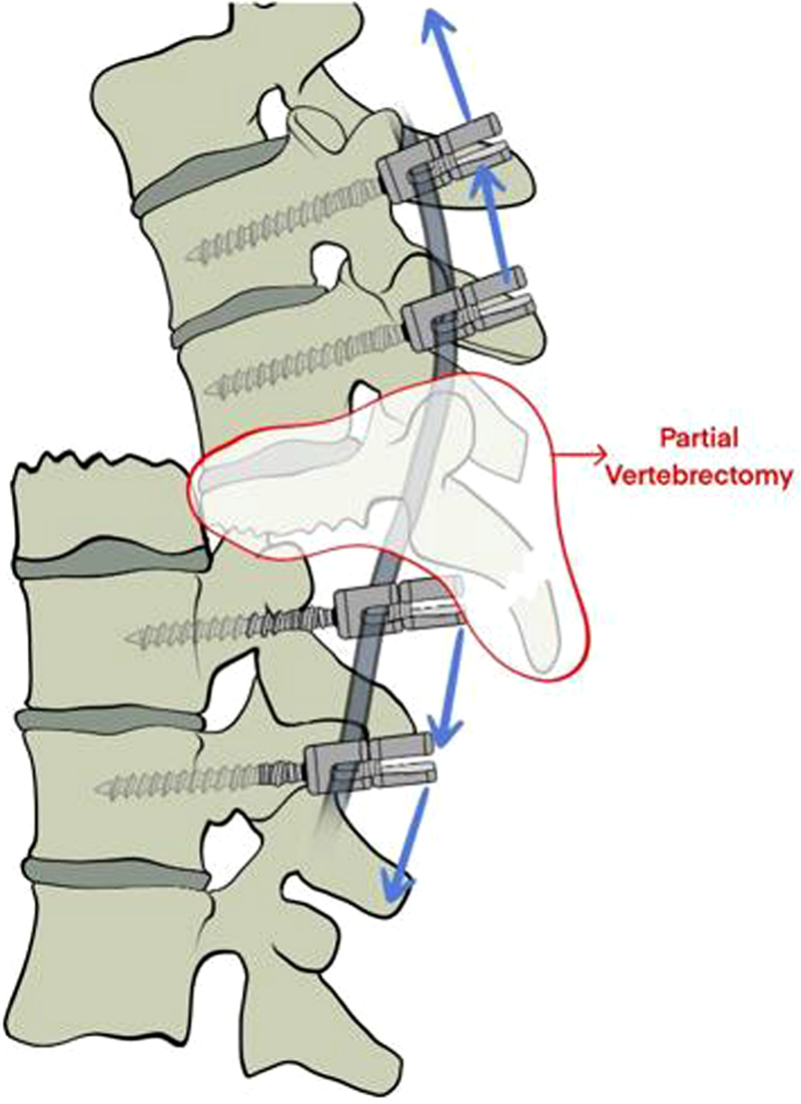
Illustration of partial vertebrectomy marking the resection. Eight pedicle screws with long tails were inserted into T10–L2. A temporary rod was inserted transversely between the pedicle screws at the level above and below the dislocation. The upper vertebral body of posteriorly dislocated T12 and its overlying disc were removed completely using rongeurs and forceps in a piecemeal fashion to facilitate distraction (as indicated by the red curve). The rods with appropriate anatomic sagittal profile were introduced first to the superior pedicle screws and the caudal part of the spine was reduced by means of submitting the 2 rods into the inferior screw heads which were purposely pre-contoured. The remaining lower approximate 2/3 part of the T12 body and the inferior endplate of T11 was curetted until the cancellous bone was well exposed for arthrodesis.

Before proceeding, the circumferential release of fibrous scarring and careful subperiosteal dissection around the affected vertebra (T12) to the anterior margin were accomplished with special attention to the vascular structures anterior to the caudal spine, after a rib resection and separation to serve the spinal-shortening maneuver. The upper vertebral body of the posteriorly dislocated T12 and its overlying disc were removed completely using rongeurs and forceps in a piecemeal fashion to facilitate distraction, namely, a partial vertebrectomy as illustrated in Figure 2, marking the resected parts. The remaining lower approximately two-thirds of the T12 body and the inferior endplate of T11 were curetted until cancellous bone was well exposed for arthrodesis. After the partial vertebrectomy procedures, the reduction by means of a rod distraction was straightforward. Specifically, the rods with an appropriate anatomic sagittal profile were introduced first to the superior pedicle screws, and the caudal part of the spine was reduced by means of submitting the two rods into the inferior screw heads, which were purposely pre-contoured. Bilateral augmented fenestration and decompression of T11 nerve roots were carried out considering a possible neurological recovery. The thecal sac was then repaired using a running 6–0 Prolene suture. Regular spinal fusion procedures were completed between T11 and the remaining T12. Adequate reduction was confirmed by intraoperative fluoroscopy. The operative blood loss was approximately 950 ml and the operation time was 195 min.

**Figure 3:**
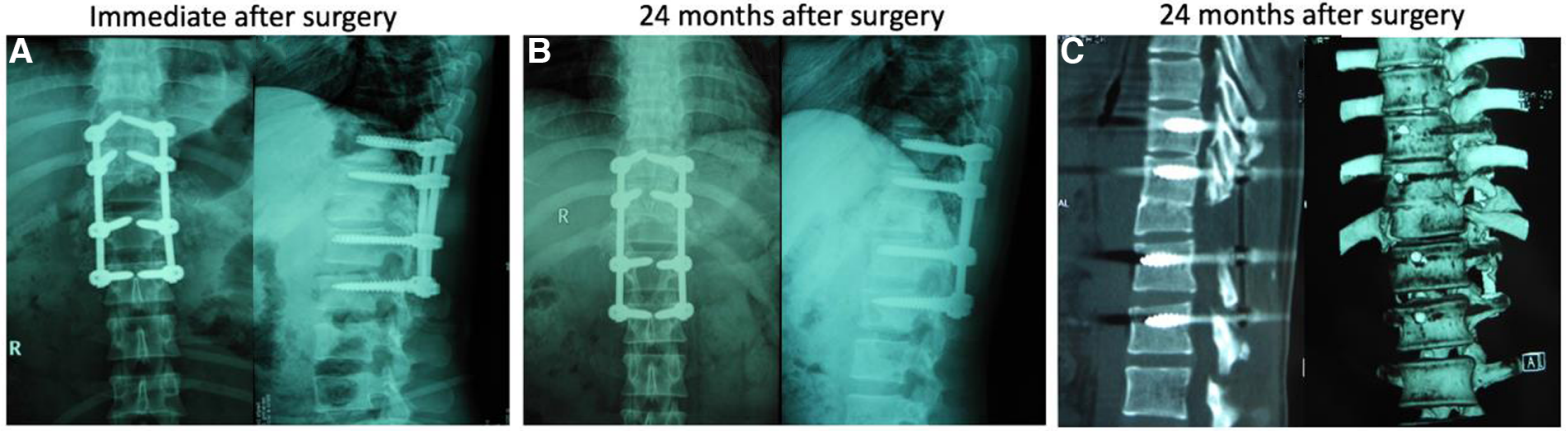
Immediate x-rays (**A**) after surgery showed satisfactory reduction of T12 dislocation by partial T12 vertebrectomy and spine shortening, with T10–L2 instrumentation and T11–12 spinal interbody fusion; At 24 months follow-up, x-rays (**B**) and CT scans (**C**) demonstrated that the good reduction was maintained and excellent interbody fusion at the osteotomy site was achieved.

The patient experienced a smooth recovery after the operation. The postoperative X-rays depicted a satisfactory reduction with good contact between T11 and the remaining T12 (Figure 3A). He was allowed to sit up with a chest brace on postoperative day 8 and was discharged to a rehabilitation unit 9 days after the operation. At 12 months of follow-up, the patient was almost free of back pain. At the last follow-up, he regained personal independence by driving a motor vehicle with hand control. The good reduction was sustained (Figure 3B) and solid interbody fusion was confirmed on reconstructive CT (Figure 3C). However, his neurological deficit at the T12 level was unchanged throughout the 2-year follow-up period.

## Discussion

A perusal of related literature in English revealed there are no reports specifically addressing old spondyloptosis at the thoracolumbar spine using partial vertebrectomy and spinal shortening. The surgical outcomes of this retrospective clinical study demonstrated that partial vertebrectomy and spinal shortening are of excellent feasibility to reduce delayed spondyloptosis via a one-stage posterior approach. The intraoperative data showed its efficacy and safety. All patients acquired satisfactory realignment and immediate stabilization of the spine without causing iatrogenic injuries to viscera injuries. Bellew et al. (3) reported a dramatic neurological recovery with a delayed correction of traumatic L2–3 spondyloptosis. Wang et al. (10) also noted a neurological improvement in 2 of 11 patients after a reduction of thoracolumbar spondyloptosis. Excitingly, this study also showed a neurological improvement of one ASIA grade in two patients. Although most patients (six out of eight) sustained paraplegia, they were able to sit up in a wheelchair with minimal to no lower back pain. Furthermore, all cases achieved spinal fusion and maintained alignment of the spinal column. Our cases confirmed the data from Alobaid et al. (11) that long-segment instrumentation is the cornerstone to maintaining postoperative stabilization and subsequent successful spinal fusion. In summary, all the above results demonstrated the clinical value of a partial vertebrectomy with spine shortening in the management of old thoracolumbar spondyloptosis.

Complete fracture-dislocation at the thoracolumbar spine is a rare occurrence, with only sporadic reports in English. A major challenge in cases with thoracolumbar spondyloptosis is how to achieve reduction. Good reduction depends on adequate distraction of dislocation space to unlock the overriding vertebrae and bring the spinal elements out to their length. For a spinal injury, posterior decompression is most effective in the documented 3- or 5-day window. Sekhon et al. (12) presented a case with traumatic T12–L1 spondyloptosis undergoing a successful posterior reduction and stabilization via a single-stage posterior approach. Although the fragments and fracture ends are mobile and amenable to intraoperative distraction at the early stage, general operations intended for early spondyloptosis become difficult in cases of delayed intervention. Because scarring and adherences wrapping the fracture site developed over time, a classic distraction and reduction was blocked and it was easy to incur additional complications secondary to improper operation. Wang et al. (10), in a study of retrospective case reports, introduced their experience in manipulating a thoracolumbar fracture-dislocation and described that fracture-dislocation delayed more than 1 week after injury was more difficult to be distracted compared to a fresh fracture-dislocation.

There were no guidelines on how to schedule a surgical strategy in cases with old thoracolumbar spondyloptosis; therefore, the authors tried to conceive a better strategy to deal with this difficult scenario. Spine shortening was an inevitable procedure to free space for spine distraction and reduction considering the abnormal bone healing and fibrous scarring. A total vertebral resection with spinal shortening was initially indicated for severe and rigid spine deformities (13) or vertebral malignancies (14, 15). In recent years, the posterior strategy by total vertebrectomy with spine shortening was frequently introduced to facilitate the reduction of spondyloptosis (16–18). Recently, Umana et al. (19) shared in a letter their initial experience with bilateral thoracic corpectomy treating traumatic vertebral fracture. However, total vertebrectomy with aggressive spine shortening seemed too invasive to patients with old spondyloptosis, factoring in their surgical tolerance. Furthermore, partial vertebrectomy was first described by Reyes-Sanchez et al. (9) in 2002 for the treatment of burst fractures and was successfully applied in other spinal diseases (20, 21). Later, Obeid et al. (22) described a novel spinal shortening technique using a one-stage posterior L5 partial spondylectomy for the management of L5–S1. This seven-case series study showed its safety and efficiency in spondyloptosis reduction. Inspired by its practicality, we, therefore, decided to apply a partial vertebrectomy with spine shortening via a posterior-only approach to manage old thoracolumbar spondyloptosis.

In our experience, comprehensive radiological evaluation using a combination of advanced modalities, such as MRI, helical 3D-CT, and X-ray imaging, should be dedicated to the operating segment of the spine in characterizing the intraoperative context. Despite this, reduction and distraction remain unexpected and challenging. First comes satisfactory reduction, essentially dependent on adequate distraction of dislocation space to unlock the overriding body of the vertebrae with the help of intraoperative traction. In patients receiving delayed surgery, the contracted soft tissue and the fibrous scar growing around the lesion would obstruct the reduction. Notably, because of the proximity of the anterior displaced vertebral body to the aorta, there is a hazardous potential for vascular injury from a forceful dorsal reduction. Posterior instrumentation without circumferential releases may result in inadequate reduction. A sample case, reported by Wang et al. (10), with complete fracture-dislocation at L1/2 received a delayed operation 28 days after injury, where anatomical reduction was not achieved. Inadequate reduction may lead to nonunion, implant failure, loss of reduction, and residual neurological compression. After adhesion release around the fracture site, the partial vertebrectomy comes forward. Based on preoperative imaging, great vessels or viscera around the dislocation site tend to shift to the anterior margin of the anteriorly dislocated column, which makes a partial vertebrectomy, resecting the posteriorly displaced part of the fractured vertebra, safer without disturbing anteriorly positioned great vessels or viscera. There are several advantages of the partial vertebrectomy-spine shortening technique over total vertebrectomy-spine shortening advocated by Obeid et al. (23) and Barcelos and Botelho (7). First, the purpose of osteotomy is to perform circumferential releases and gain adequate mobility for anatomical reduction. Removal of the dorsally displaced vertebral part and its surrounding fibrous scarring can achieve the goal, which is less traumatic and technically demanding. Second, because the course of the great vessel and the position of some viscera may change, there may be possible iatrogenic damage to them during the total vertebrectomy. Furthermore, according to the experiment by Kawahara et al. (24), the safe osteotomy range of spinal shortening is within two-thirds of the vertebral segment, which sustains the blood flow of the spinal cord and benefits neural recovery. Performing a partial vertebrectomy with less than two-thirds of the vertebral body resected leads to the spine not over-shortening and achieving better interbody fusion.

Considering the total devastation of all spinal elements around the fracture site, stabilization of the spine is believed to almost completely rely on the instrumented contract of pedicle screws and rods. In order to avoid postoperative instrumentation-related complications, such as re-dislocation and nonunion, long and strong contracts (at least two levels above and two levels below) are mandatory here to make sure there is sufficient force for stabilization and successful fusion (17, 23). Whenever possible, the upper part of the fractured vertebra after a posterior resection should also be instrumented as an intact pedicle confirmed. It should be noted that in some cases, such as the illustrative case above, the remaining lower vertebral part is not instrumented after reduction and anterior column restoration, rendering this a risk factor to spinal stability and bony fusion. There are two alternative techniques to minimize its potential impact. First, the reduction ends are technically demanded to be curetted until the cancellous bone is well exposed, thus ensuring direct and tight bone-on-bone contact between two ends under axial compression to enhance osteogenesis (25). However, this technical procedure of cancellous bone-on-bone contact instead of vertebral endplates brings an additional risk of subsidence of the vertebral bodies that are overlapped on each other, as indicated by Obeid et al. (22) in their management of L5 spondyloptosis using a partial vertebrectomy. Hereby, adequate circumferential fusion with bone grafting of autogenic bone and allogenic bone grafts is strongly suggested as the supplemental procedure to improve spinal fusion (26–28). On the other hand, the paralysis status of the patient provides more immobilization time to reduce the load from the anterior weight-bearing column than the non-paralysis patient, thus benefiting spinal fusion. Because of those technical efforts in our case series, seven out of eight patients achieved solid fusion with only one patient experiencing slight re-dislocation but ending with spinal fusion. The favorable spinal fusion result demonstrates that such an unstable situation seems to have little impact on future fusion in our case series.

In summary, preoperative considerations should include advanced imaging and a careful evaluation of the distorted anatomy and the patient’s general condition, while paying attention to the course of ventral great vessels. Intraoperatively, meticulous lysis of adhesions and extensive scar release around the fracture are essential for a subsequent safe maneuver. In addition, a partial vertebrectomy of the posteriorly dislocated part of the index vertebra combined with long-segment instrumentation (at least two levels below and two above the fracture site) is, in principle, necessary to maintain spinal stabilization and to improve spinal fusion. The extent of partial vertebrectomy mainly depends on the intraoperative contextual circumstance. Since it is very technically demanding and exhausting, meticulous surgical technique, rich experience, and extreme care are required for satisfactory outcomes.

## Conclusion

Partial vertebrectomy with spinal shortening via a posterior approach showed good efficacy and safety in managing old spondyloptosis of the thoracolumbar spine after trauma, allowing for a one-step good reduction and spinal fusion for early rehabilitation.

## Data Availability

The original contributions presented in the study are included in the article, further inquiries can be directed to the corresponding author.
